# Genome-Wide Association Study of Blood Mercury in European Pregnant Women and Children

**DOI:** 10.3390/genes14122123

**Published:** 2023-11-24

**Authors:** Kyle Dack, Mariona Bustamante, Caroline M. Taylor, Sabrina Llop, Manuel Lozano, Paul Yousefi, Regina Gražulevičienė, Kristine Bjerve Gutzkow, Anne Lise Brantsæter, Dan Mason, Georgia Escaramís, Sarah J. Lewis

**Affiliations:** 1 Medical Research Council Integrative Epidemiology Unit, University of Bristol, Bristol BS8 1TH, UK; kyle.dack@bristol.ac.uk (K.D.);; 2 ISGlobal, Institute for Global Health, 08036 Barcelona, Spain; 3 Universitat Pompeu Fabra (UPF), 08018 Barcelona, Spain; 4 Spanish Consortium for Research on Epidemiology and Public Health (CIBERESP), 28029 Madrid, Spaingeorgia.escaramis@gmail.com (G.E.); 5 Centre for Academic Child Health, Bristol Medical School, University of Bristol, Bristol BS8 2PS, UK; caroline.m.taylor@bristol.ac.uk; 6 Epidemiology and Environmental Health Joint Research Unit, FISABIO- Universitat Jaume I - Universitat de València, 46020 Valencia, Spain; 7 Department of Preventative Medicine, Food Sciences, Toxicology and Forensic Medicine Department, Universitat de València, 46100 Valencia, Spain; 8 Department of Environmental Sciences, Faculty of Natural Sciences, Vytautas Magnus University, 53361 Kaunas, Lithuania; 9 Department of Air Quality and Noise, Division of Climate and Environmental Health, Norwegian Institute of Public Health, P.O. Box 222 Skoyen, NO-0213 Oslo, Norway; kristinebjerve.gutzkow@fhi.no; 10 Department of Food Safety, Division of Climate and Environmental Health, Norwegian Institute of Public Health, P.O. Box 222 Skoyen, NO-0213 Oslo, Norway; 11 Bradford Teaching Hospitals NHS Foundation Trust, Duckworth Lane, Bradford BD9 6RJ, UK; 12 Department of Biomedical Sciences, Institute of Neuroscience, University of Barcelona, 08035 Barcelona, Spain; 13 Population Health Sciences, Bristol Medical School, University of Bristol, Bristol BS8 1TH, UK

**Keywords:** ALSPAC, HELIX, blood mercury, GWAS, pregnancy, children

## Abstract

Mercury has high industrial utility and is present in many products, and environmental contamination and occupational exposure are widespread. There are numerous biological systems involved in the absorption, metabolism, and excretion of Hg, and it is possible that some systems may be impacted by genetic variation. If so, genotype may affect tissue concentrations of Hg and subsequent toxic effects. Genome-wide association testing was performed on blood Hg samples from pregnant women of the Avon Longitudinal Study of Parents and Children (*n* = 2893) and children of the Human Early Life Exposome (*n* = 1042). Directly-genotyped single-nucleotide polymorphisms (SNPs) were imputed to the Haplotype Reference Consortium r1.1 panel of whole genotypes and modelled againstlog-transformed Hg. Heritability was estimated using linkage disequilibrium score regression. The heritability of Hg was estimated as 24.0% (95% CI: 16.9% to 46.4%) in pregnant women, but could not be determined in children. There were 16 SNPs associated with Hg in pregnant women above a suggestive *p*-value threshold (*p* < 1 × 10^−5^), and 21 for children. However, no SNP passed this threshold in both studies, and none were genome-wide significant (*p* < 5 × 10^−8^). SNP-Hg associations were highly discordant between women and children, and this may reflect differences in metabolism, a gene–age interaction, or dose–response effects. Several suggestive variants had plausible links to Hg metabolism, such as rs146099921 in metal transporter SLC39A14, and two variants (rs28618224, rs7154700) in potassium voltage-gated channel genes. The findings would benefit from external validation, as suggestive results may contain both true associations and false positives.

## 1. Introduction

Environmental Hg concentrations have increased substantially over the past century, and this is largely attributed to human industrial activity [1]. Rising emissions from human activity is expected to lead to increased human exposure to Hg over the coming decades [2], and the health and economic costs are projected to be considerable [3]. There are three common forms of Hg found in the human environment—elemental, inorganic (I-Hg), and the organic compound methylmercury (MeHg) [4]—each of which is highly toxic [5].

Human exposure to environmental Hg is possible under a variety of circumstances, but in most populations occurs primarily through dietary consumption of I-Hg and MeHg. Emissions of elemental and I-Hg are released as a by-product of industrial processes such as coal burning, and these emissions are dispersed globally into the atmosphere, oceans, and soils [6,7]. Hg deposits in the ocean shallows can be methylated by microorganisms to form MeHg [8]. This organic compound tends to remain stable within organisms which leads to accumulation up the food chain in increasing concentrations, with the greatest concentrations of MeHg observed in long-living predatory fish such as swordfish, tuna, and king mackerel [9,10]. A major source of human exposure is the consumption of these fish [11], in addition to other potentially contaminated foods such as rice, cereals [12,13], and meat [14], and trace quantities are possible in other food products [11]. Apart from diet, exposure may occur in occupations which involve handling Hg [15], or among those who have silver amalgam fillings, smoke cigarettes, or use skin-whitening creams or traditional medicines which may be contaminated with Hg [16].

After absorption, I-Hg tends to bind to thiol-containing proteins, and is quickly transported by plasma proteins, such as albumin, out of circulation and into tissues throughout the body [17]. Most I-Hg is deposited into the kidneys, and only a small fraction of total exposure may be detected by blood measurements [18]. MeHg, on the other hand, binds to erythrocytes, potentially by binding to the erythrocyte membrane, or through other transport mechanisms such as those involving D-glucose or cysteine [17,19]. It is then distributed to all tissues, including the brain and placenta [20]. Most Hg measured in whole blood consists of MeHg, and therefore total blood Hg is commonly considered indicative of recent dietary MeHg exposure [21].

Hg toxicity is enabled or enhanced due to structural similarity to common amino acids [22], which facilitates binding with sulfhydryl group organic compounds, lipids, proteins, and enzymes. The toxic effects of Hg are therefore broad and can disrupt cellular functioning and tissue health throughout the body [23], leading to increased reactive oxygen species and oxidative stress [24,25], carcinogenesis [26], epigenetic changes [27], and cell death [28].

Health effects from high doses of exposure, such as from occupational accidents, can result in acute poisoning, and require immediate treatment to avoid organ failure, neurological impairment, and long-term harm [29,30,31]. However, for people who are not at risk of occupational exposure, there may still be long-term health risks from continuous dietary exposure. This is because Hg may be absorbed in greater quantities than can be excreted, leading to increasing concentrations of Hg accumulated in tissues. Hg is slow to clear from the body due to cycles of methylation and demethylation between the forms of Hg which alter metabolic pathways [32] and the tendency for Hg in the kidneys or intestines to be reabsorbed into circulation [18,33]. For these reasons, the biological half-life corresponding to an approximate halving of internal concentrations is 1–3 weeks for I-Hg [34], and several months for MeHg [35]. Environmental Hg exposure is associated with increased blood pressure [36], risk of heart disease [37], kidney disease [38], and a wide range of neurological symptoms [21,39]. Pregnant women are a particularly vulnerable population, because Hg can readily cross the placenta [40] and accumulate at a higher Hg-to-weight ratio in the developing infant. However, the evidence for an effect of mercury in the general population on detectable developmental impairment is currently mixed [41,42].

Considering the complex pathways of absorption, transport, tissue distribution, and excretion, it is likely that genetic variation may mediate the relationship between environmental Hg and human exposure. Studies have reported associations between single-nucleotide polymorphisms (SNPs) in genes related to Hg metabolism and internal levels of Hg, such as in the glutathione metal-binding detoxification system [43,44,45], the metallothionein metal transport family [46], lipid-transport protein apolipoprotein-E [47], and genes involved in iron homeostasis [48]. Hg interacts with other elements such as selenium [49], zinc [50,51], cadmium, and lead [52,53], and variants which alter concentrations of these elements may also impact Hg [54,55,56]. The utility of identifying SNP-Hg associations is twofold; first, it can enhance our understanding of the biological mechanisms of how Hg acts on the body, second, it may enable new methods to test the impact of Hg on health by using SNPs as randomised proxies of Hg exposure [57].

Prior studies have tested associations between Hg and SNPs in genes targeted for their theoretical relevance. However, genome-wide association testing may be advantageous in identifying a greater proportion of relevant SNPs. This approach is hypothesis-free and all available SNPs are tested, so variants can be identified not only in genes but also in non-coding regions of the genome [58,59]. This method has previously been used to identify novel genetic variants associated with blood concentrations of copper [55], iron [60], lead [54], manganese [61], selenium [55,56], and zinc [55].

The objective of this study was to assess the associations between SNPs and blood Hg concentrations in pregnant women and children using genome-wide association testing. Specific aims were (1) to estimate the heritability of blood Hg levels using linkage disequilibrium score (LDSC) regression, (2) to perform genome-wide association testing between imputed SNPs and blood Hg in two European populations, (3) to explore the function of strongly associated SNPs through in silico analyses, and (4) to compare associations between Hg and candidate variants identified from previous studies.

## 2. Materials and Methods

### 2.1. Overview

Genome-wide associations were estimated between SNPs and blood Hg concentrations in two separate European studies, one of pregnant women and one of children. Table 1 includes a brief summary of the characteristics of each study, with more details available in Supplementary Table S1.

### 2.2. The Avon Longitudinal Study of Parents and Children (ALSPAC)

ALSPAC is a multi-generational birth cohort in the former Avon Health Authority area in the UK. All pregnant women living within this area with expected dates of delivery between 1 April 1991 and 31 December 1992 were invited to take part in the study. From 20,248 pregnancies identified as eligible, 14,541 were initially enrolled, which, after accounting for multiple pregnancies, resulted in 14,203 unique mothers. This was expanded with additional phases of recruitment to provide a total of 14,833 unique women in the study. Full details of the recruitment process and sample profile are described elsewhere [62,63]. Details of all the data that are available from the study are available in a fully searchable online data dictionary and variable search tool: http://www.bristol.ac.uk/alspac/researchers/our-data/ (accessed on 1 October 2023). Participant characteristics were representative of most UK women. However, women were predominantly of European ancestry, and due to the potential for population stratification, only European women were included in this GWAS analysis, which limits the generalizability of findings to populations with different ancestry [62].

Whole blood samples were taken from 4844 pregnant women during early antenatal care visits, with a median visit time of 11 weeks of gestation (IQR: 4 weeks). A vacutainer system was operated by midwives to draw the samples, which were stored at 4 °C for 1–4 days before being sent to the central Bristol laboratory. Samples were transported for up to 3 h at room temperature, and then stored at 4 °C until the time of analysis.

Whole blood Hg was measured using inductively coupled plasma dynamic reaction cell mass spectrometry (ICP-DRC-MS) at the Centers for Disease Control and Prevention (CDC), Bethesda, CDC method 3009.1. Quality control (QC) measures are described in earlier studies [55,64], which left 4131 measurements after exclusions. One sample was below the limit of detection for Hg (0.24 μg/L) and was assigned a value 0.7 times the lower limit of detection [65].

Blood samples for DNA analysis were taken during pregnancy from 10,015 women [66]. Samples were genotyped by Centre National de Génotypage (CNG) using the Illumina Human660W-Quad Array. Genotype annotation was performed using Illumina GenomeStudio [67], and aligned to GRCh37 with the software Burrows-Wheeler Aligner version 1. QC procedures were applied to the genotyped data using Plink v1.07 [68]. SNPs were excluded if they were missing from more than 5% of individuals, had a Hardy–Weinberg Equilibrium (HWE) *p* < 1.0 × 10^−7^, or a minor allele frequency (MAF) of less than 1%. Individuals were excluded if they were missing more than 5% of SNPs, had indeterminate X chromosome heterozygocity or extreme autosomal heterozygocity (>3 standard deviations from population mean), were population outliers using four HapMap populations as a reference, or had a cryptic relatedness estimate equivalent to first cousin or closer (identify by descent, IBD > 0.125) with another individual in the sample [69,70]. Directly genotyped SNPs were imputed to the Haplotype Reference Consortium (HRC r1.1) panel of approximately 31,000 phased whole genotypes. Phasing was performed using ShapeIt v2 [71] and imputation using Impute V3 on the Michigan Imputation Server [72]. SNPs were excluded following imputation where MAF <1% or imputation quality score (INFO) < 0.9.

### 2.3. The Human Early Life Exposome (HELIX)

HELIX comprises subcohorts of mother–child pairs from six European birth cohorts [73,74]. The cohorts enrolled approximately 32,000 pairs between 1999 and 2010 in the UK, France, Spain, Lithuania, Norway, and Greece [75] (Table 2). From these studies, 1301 children were included in the HELIX subcohort, which measured a variety of pre- and postnatal exposures, health outcomes, and genome-wide genotypes. The current study only included children with genetic data, Hg levels, and who were of European ancestry (determined from genome-wide genetic information) (*n* = 1042).

Child blood samples were collected during follow-up clinic visits between December 2013 and February 2016, when the children were aged 6 to 11 years old [76]. All cohorts followed the same procedures and analysis protocols. Whole blood was stored in EDTA vacutainers and analysed for trace element testing and DNA extraction at ALS Scandinavia (Sweden). Total Hg levels were measured using double focusing sector field inductively coupled plasma mass spectrometry (ICP-SFMS) as described elsewhere [77]. The limit of detection was 0.02 µg/L.

The Infinium Global Screening Array (GSA) (Illumina) was used for genome-wide genotyping at the Human Genomics Facility (HuGe-F), Erasmus MC (www.glimdna.org). GenomeStudio 2.0 software with the GenTrain2.0 algorithm was used for genotype calling, and annotation on GRCh37 using the GSAMD-24v1-0_20011747_A4 manifest. Samples were excluded if there was SNP missingness >3%, sex mismatch, heterozygosity (>4 SD), cryptic relatedness (Pi-hat > 0.185), or duplicates. SNPs were excluded if missing from >5% individuals, MAF < 1%, or HWE *p* < 1.0 × 10^−6^.

Genotype information was phased to HRC r1.1 using Eagle v2.4 and imputed using Minimac4 and the Michigan Imputation Server [78]. Post-imputation filtering was applied to exclude SNPs with low imputation quality (R^2^ < 0.9), allele frequency (MAF < 1%), or HWE *p* > 1.0 × 10^−7^.

### 2.4. SNP Heritability

Heritability refers to the amount of outcome variation which is attributable to genetic differences. We estimated Hg heritability for measured SNPs (h^2^_g_) by applying LD score regression [79,80] to summary statistics from each GWAS. In brief, this method involved taking SNP-level data and regressing standardised SNP-Hg associations on the sum of correlations between a SNP and those nearby, known as LD scores. The rationale behind this is that a high LD score increases the probability that a SNP is correlated with a true causal SNP of Hg. LD scores were taken from a reference panel computed from 1000 Genomes Project European data [81], which was accessed from https://data.broadinstitute.org/alkesgroup/LDSCORE (accessed on 1 October 2023) with the filename ‘eur_w_ld_chr’.

### 2.5. Genome-Wide Association Testing

Genome-wide association testing was performed to estimate the association between each SNP and a continuous Hg phenotype. In ALSPAC, the GWAS of women was conducted in SNPTEST version 2.5.2 using the frequentist option and “score” method of accounting for genotype uncertainty [82]. In HELIX, the GWAS of children was run using PLINK version 1.0 with the “--linear” option [68]. Each analysis was adjusted for age at the time when blood was taken and eigenvectors for the first 10 principal components estimated from GWAS data. The continuous linear models assumed an additive effect of SNPs and a normal distribution of phenotype residuals. The distribution of Hg had a strong right skew and if regressed in its raw form was unlikely to meet the latter assumption, and this could bias standard error and *p*-values. To address this, Hg measurements were log2-transformed to approximate a normal distribution.

Follow-up analysis was performed in R version 4.1.0 unless otherwise stated. Reference SNP IDs were missing from all ALSPAC results and some HELIX, and therefore chromosome and location were used to identify labels valid for GRCh37 using the “SNP locations for Homo sapiens (dbSNP Build 144)” reference table and “BSgenome” R packages [83,84]. Results were visualised with quantile–quantile (QQ) and Manhattan plots generated using the “qqman” package [85]. SNPs were classified as genome-wide significant if *p* < 5 × 10^−8^, and suggestively significant if p was between 1 × 10^−5^ and 5 × 10^−8^. Variants in linkage disequilibrium (R^2^ > 0.1 and 250 kb range) were grouped using the ld_clump function of the MRC IEU GWAS R package [86] and the most significant SNP kept. The strongest results from each GWAS were compared.

In addition to identifying suggestive and significant SNPs, summary statistics were extracted and reported for 13 variants of interest which were previously identified as (a) associated with Hg levels in candidate gene studies or (b) associated with metals which may interact with Hg levels in genome-wide association studies (Supplementary Table S2).

### 2.6. In Silico Functional Analysis

All variants with *p* < 1 × 10^−5^ were mapped to the nearest gene using the SNP2Gene function in Functional Mapping and Annotation of Genome-Wide Association Studies (FUMA) [87], and the results were verified in NCBI Sequence Viewer [88]. The potential biological mechanisms of how the variants may affect Hg were investigated using tools which aggregated prior genetic research. SNP-phenotype associations were explored using FUMA eQTL [87], LDtrait [89], and PhenoScanner [90,91]. Gene functions were explored in the GeneCards database [92], gene–tissue expression using the GTEx portal [93,94], and gene–phenotype associations in the Online Mendelian Inheritance in Man database [95].

## 3. Results

### 3.1. Study Characteristics

**Figure 1:**
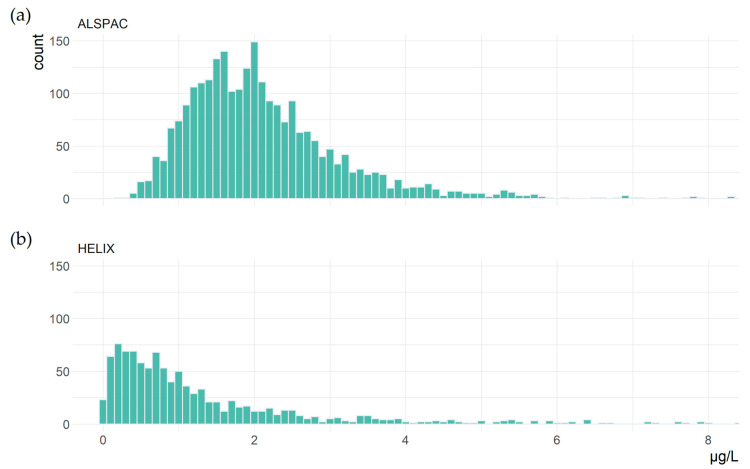
Blood Hg concentrations in (**a**) 2890 women and (**b**) 1038 children aged 6–11 years old.

The derivation of the number of participants and SNPs included in each study is shown in Table 3. There were 2893 women included in this study, with a median age of 28.0 years (IQR: 6.0). The mean concentration of blood Hg was 2.09 μg/L (standard deviation, SD: 1.08) and median 1.89 μg/L (IQR: 1.16). The study included 1042 children with a median age of 8.0 years (IQR: 2.4) and 54.6% were male. As seen in Figure 1, Hg concentrations were lower for children, with mean Hg 1.35 μg/L and median 0.82 μg/L, and there was slightly more variance (IQR: 1.27). For improved readability, Figure 1 excludes seven samples where Hg > 8 μg/L, and complete histograms are available in Supplementary Figure S1.

### 3.2. SNP Heritability

The SNP heritability (h^2^_g_) of Hg was calculated from summary statistics of the GWAS performed in this study. Data were available for 6,620,135 and 6,138,843 SNPs for women and children, respectively. Standardised effect estimates were regressed on LD scores of each SNP taken from the 1000 Genome Project Europeans reference panel. Merging summary statistics and the reference panel resulted in 1,137,154 and 965,135 SNPs for women and children available for LD score regression.

The estimated h^2^_g_ for women was 24.0% (95% CI: 16.9% to 46.4%, *p* = 0.01). For children, the estimate was 4.8%, but the evidence for this was very weak (*p* = 0.85), and confidence intervals were very wide and overlapped with zero (95% CI: −45.7% to 55.4%).

### 3.3. Genome-Wide Association Testing

SNP-Hg associations are visualised in Manhattan plots in Supplementary Figure S2, and expected and observed *p*-values are compared in Supplementary Figure S3 using QQ plots. The plots did not show visible inflation or deflation of *p*-values, and this was reflected in lambda statistics of 1.01 for women and 1.00 for children.

No SNPs were found to be genome-wide-significant (*p* < 5 × 10^−8^) for women or children. At a lower threshold of *p* < 1 × 10^−5^, there were suggestive associations at 16 independent loci for women and 21 for children. The strongest association at each loci was selected, and summary statistics are presented in Table 4.

In total, there were 37 SNPs at independent genetic loci with suggestive associations in one of the studies. However, none of these were found to be associated in the alternative study even at a more relaxed threshold of *p* > 1 × 10^−2^. Not all SNPs could be compared between studies, due to presenting at low MAF (lower than the threshold for inclusion) in one of the cohorts.

### 3.4. In Silico Functional Analysis

The 37 SNPs with suggestively significant associations with blood Hg were mapped to their nearest genes using NCBI Sequence Viewer and FUMA SNP2Gene (Supplementary Table S3). Additionally, we identified variants strongly associated with the expression of other genes (*p* > 5 × 10^−8^) using Phenoscanner, and included those genes in the following analyses.

SNPs were most commonly associated with gene expression, histone modification, and methylation at genes or CpG sites close to the SNP locations (Supplementary Table S4). No SNPs or strongly correlated SNPs (r^2^ > 0.8) were found to have direct evidence of connections to Hg metabolism, but several were located inside genes with potential links to Hg.

In this study, the T allele of intronic variant rs146099921 was associated with −0.12 log Hg (*p* = 8.21 × 10^−6^) for women. This SNP is located in the gene Solute Carrier Family 39 Member 14 (*SLC39A14*), and within the gene is associated with DNA methylation at cg14348540 [96,97] and exon expression [98]. *SLC39A14* (also referred to as *ZIP14*) is a metal transporter linked to cellular uptake of cadmium, iron, manganese, and zinc [99]. Mutations are associated with the impairment of manganese transport and homeostasis, leading to toxic accumulation [100,101,102]. There is evidence that the gene functions as a transporter of zinc [103], and mediates iron and cadmium uptake [104,105], and a detailed review of transport functions was identified [99]. According to data available in the GTEx Portal [94], *SLC39A14* is expressed most highly in the liver, followed by the adipose tissue, the arteries, and pancreas.

There were further links between suggestive variants and genes with functions potentially affecting Hg levels, such as for rs17106291 (Solute Carrier Family 25 Member 21, *SLC25A21*) which transports dicarboxylates across the inner membranes of mitochondria [106]. Two variants (rs28618224, rs7154700) were in potassium voltage-gated channels genes, and two variants were near to genes affecting glutamate (rs361166) and phospholipid (rs115812569) transport.

### 3.5. Associations in Previous Candidate Variants

GWAS summary statistics were extracted for 14 SNPs which were identified a priori as variants of interest due to prior studies reporting associations with Hg or metals which interact with Hg (Supplementary Table S2). Associations with blood Hg are shown in Table 5. Most were not replicated in either women or children. Exceptions were the minor C allele of rs10636 which was associated with increased blood Hg in women (*p* = 0.01), and the minor C allele rs9936741 associated with lower Hg in children (*p* = 0.01). Neither association was replicated in the alternative study, and no association was found in variants previously reported as genome-wide-significant from GWAS of blood lead, selenium, or zinc.

## 4. Discussion

Genome-wide association testing of blood Hg from British pregnant women and children across Europe did not identify strong associations with any imputed SNPs. Despite this, heritability from approximately 1 million SNPs was estimated to explain a considerable proportion of Hg variance in pregnant women (24.0%, 95% CI: 16.9 to 46.4). Considering that Hg is highly reactive with a wide range of molecules and exposure is affected by numerous biological processes, it is expected that a substantial component of its metabolism would be heritable and the finding is consistent with animal and plant studies [109,110] which also estimated there were large genetic components to Hg variation.

Although no variants passed genome-wide significance thresholds, there were 37 independent loci detected between the two studies at a lower threshold considered suggestive of an association. Surprisingly, no SNP was found to be suggestively associated in both women or children, even at a more relaxed threshold of *p* > 1 × 10^−2^. There are several possible reasons for this. First, there may be qualitative differences in Hg metabolism between pregnant women adults and children. It is possible that metabolic processes change with age or pregnancy, and although no human studies have explored or speculated on this, there is evidence in animals that rates of Hg absorption and excretion are different in early life compared to adulthood [111,112]. Secondly, the child GWAS was much smaller (1042 vs. 2893) and median blood Hg level was lower (0.82 vs. 1.89 μg/L), which may have led to a lack of statistical power, increased rates of false positives, and/or reduced SNP effects due to lower concentrations of Hg. We found indications of this in our heritability analyses, where there was insufficiant certainty to produce a meaningful estimate (h^2^_g_ = 4.8%, 95% CI: −45.7% to 55.4%). A final reason for the heterogeneity may be if non-linear associations exist between SNPs and Hg, if for example a gene is only expressed above a certain threshold of Hg exposure.

The SNP rs146099921 was identified as the most biologically plausible of those with suggestive associations with Hg, located in the gene *SLC39A14*. While the variant is intronic, there is evidence it has an active effect through modification of *SLC39A14* gene expression [98], methylation [96,97], and exon expression [98]. Most SLC39 genes are responsible for the cellular uptake of zinc [113], but studies suggest *SLC39A14* is associated with multiple metals, including cadmium, iron, and manganese levels [99,100,104]. It is possible the gene impacts Hg levels indirectly through these metals, each of which may interact with Hg—for example, increased cellular zinc induces metallothionein synthesis which may promote removal of Hg [51]. Alternatively, *SLC39A14* may directly affect the transport of Hg, but this does not appear to be reported in prior studies. In children, a suggestive association was found with rs17106291, which is located in *SLC25A21*, a transporter of C5-C7 oxodicarboxylates to mitochondria. Prior studies have linked other members of the *SLC* gene family to kidney uptake of I-Hg [114] and the intestinal transport of MeHg [17].

Several other suggestive SNPs were annotated to genes with possible connections to Hg metabolism. Associations were identified in SNPs located in potassium voltage-gated channels genes *KCNH5* and *KCNIP4* and the calcium ion channel gene *TRPC4*. These are potentially relevant to Hg, because expression of MeHg and I-Hg toxicity may be linked to inhibited potassium or calcium channels [51], although it is unclear what impact would be expected on blood Hg. Finally, there were variants located in proximity to genes affecting glutamate (*GLS*, *GATB*), relevant because Hg both inhibits glutamate uptake [115] and stimulates its release [116].

Associations reported in previous candidate gene studies of Hg were for the most part not replicated by this study. There were seven variants of interest to Hg metabolism, located in genes *GCLM*, *GCLC*, *TF*, *MT1A*, *MT1M*, and *MT2A*. In women, there was evidence of an association between blood HG and rs10636 (*MT2A*), and the direction of effect was consistent with that previously reported [46]. In children, rs9936741 (*MT1M*) was associated with Hg but in the opposite direction than a prior study [46], potentially due to the use of hair Hg in the original study. Both variants have a biologically plausible link to Hg: metallothionein generates proteins which bind to Hg toaid clearance [117]. Neither of the above associations were replicated in the alternative study, and the *p*-values of each association (*p* = 0.01) provide only tentative evidence because they were considerably below genome-wide thresholds. However, these variants were selected a priori and it therefore seemed more appropriate to apply standard observational thresholds. No other candidate SNP associations were replicated. This may be due to the smaller sample sizes used in prior studies which increased the likelihood of spurious results, or due to differences in tissues used to measure Hg. Finally, an epigenome-wide analysis of umbilical cord Hg identified associations in the genes *GGH*, *MED31*, and *GRK1* and DNA methylation [118]. The direction of causality is unclear, but in this study no strong signals were found in variants near the reported CpG sites.

Limitations of this study were as follows. First, the lack of genome-wide significant SNPs and high heterogeneity between studies suggests one or more of the analyses may have been underpowered. By comparison, two larger GWAS reported one loci associated with blood lead levels (*n* = 5433) [54] and two with selenium levels (*n* = 9639) [56]. The required sample size is also a function of the trait variance, as demonstrated in an arsenic GWAS in Bangladesh which identified five independent loci from 1313 arsenic-exposed individuals [119]. The low power may be part of the reason for the discordance between ALSPAC and HELIX results, and in particular for SNPs with low MAF.

A second limitation is the use of blood Hg. This reflects relatively short-term exposure and is therefore subject to daily variation depending on diet, metabolism, and random noise [120,121]. In linear models, the measurement error between blood Hg and underlying exposure to Hg may lead to residual error and reduce study power. There are samples such as hair and nails which represent Hg exposure over a longer time-frame of several months, and this is something future studies should consider.

A third limitation is that the HELIX study comprised six subcohorts located in different countries. These populations had different profiles of Hg exposure and environmental variation, and while no heterogeneity was detected in the pooled analysis, it is possible this increased uncertainty in the GWAS estimates. Finally, this study was conducted on European ancestry populations, and populations with different ancestries are likely to have different genotype-Hg associations, thus our results may not be relevant to other populations.

## 5. Conclusions

In this GWAS of women and children, no SNPs were found to be associated with Hg above genome-wide significant thresholds. However, in women, SNP heritability was estimated to be around 24%, and some SNPs, in particular the variant *rs146099921*-located metal transport gene *SLC39A14*, were suggestively associated with blood Hg. Low correlations between results from pregnant women and children could reflect developmental changes in Hg metabolism, exposure levels or population heterogeneity.

## Figures and Tables

**Table 1:** Summary of included studies.

	The Avon Longitudinal Study of Parents and Children	The Human Early Life Exposome
Abbreviation	ALSPAC	HELIX
Date of recruitment	April 1991–December 1992	1999–2010
Population	Pregnant women	Children aged 7 to 9
Location	Former Avon Health Authority area, UK	UK, France, Spain, Lithuania, Norway, and Greece
Total participants	14,833	Approximately 32,000
Hg source	Whole blood	Whole blood
Hg sample timing	Early pregnancy	6–11 years old
Hg analysis method	ICP-DRC-MS	ICP-SFMS
Genotyping method	Illumina Human660W-Quad Array	Infinium Global Screening Array (GSA) (Illumina)
Imputation panel	Haplotype Reference Consortium (HRC r1.1)	Haplotype Reference Consortium (HRC r1.1)

**Table 2:** Locations of the study populations.

	Country	Sample Size
**Children (HELIX)**	-	1042
Born in Bradford (BiB)	United Kingdom	90
Étude des Déterminants pré et postnatals du développement et de la santé de l’ENfant (EDEN)	France	135
INfancia y Medio Ambiente (INMA)	Spain	198
Kaunas birth cohort (KANC)	Lithuania	196
The Norwegian Mother, Father and Child cohort study (MoBa)	Norway	237
The Rhea Mother–Child Cohort in Crete (Rhea)	Greece	186
**Pregnant women (ALSPAC)**	United Kingdom	2983

**Table 3:** Study size derivation.

	ALSPAC		HELIX	
	SNP	N	SNP	N
Direct genotyped	557,124	10,015	692,367	1397
Direct genotyped after QC ^1^	526,688	8196	509,344	1304
Imputed (HRC r1.1) ^2^	39,117,141	8196	40,405,505	1304
Imputed after QC	6,649,782	8196	6,143,757	1304
Blood Hg measurements	-	4014	-	1301
GWAS	6,649,782	2893	6,143,757	1042
GWAS after QC	6,620,135	2893	6,138,843	1042

^1^. Quality control filters; see Section 2.2 and Section 2.3. for exclusions. ^2^. Haplotype Reference Consortium panel version 1.1.

**Table 4:** Summary statistics for the variants suggestively associated (*p* < 1 × 10^−5^) with blood Hg concentrations, pruned to the most significant SNP per independent genetic loci.

Pregnant Women, ALSPAC (*n* = 2893, nSNP = 6,620,135)										
SNP	Chr	Position ^1^	Gene ^2^	Effect Allele	Other Allele	MAF ^3^	β	SE ^4^	*p*-Value	HELIX *p*-Value ^5^
rs4853739	2	191698516	*-*	T	C	0.24	0.10	0.02	2.32 × 10^−6^	-
rs11709754	3	149615531	*RNF13*	T	A	0.21	−0.10	0.02	9.39 × 10^−6^	-
rs361166	4	152792791	*-*	A	G	0.41	0.09	0.02	5.78 × 10^−6^	0.53
rs6859392	5	63241481	*-*	G	C	0.30	−0.09	0.02	3.31 × 10^−6^	-
rs1372504	5	103749428	*RP11-6N13.1*	A	G	0.38	−0.09	0.02	1.70 × 10^−6^	0.53
rs2246509	6	156058999	*-*	G	A	0.46	0.08	0.02	8.69 × 10^−6^	0.98
rs1845418	7	24095415	*-*	T	C	0.14	0.14	0.03	8.68 × 10^−8^	0.48
rs146099921	8	22254947	*SLC39A14*	G	T	0.14	−0.12	0.03	8.21 × 10^−6^	0.14
rs7900717	10	122034207	*-*	C	T	0.25	−0.09	0.02	6.02 × 10^−6^	0.31
rs7301395	12	116402976	*MED13L*	A	G	0.02	0.33	0.07	5.06 × 10^−6^	-
rs12874443	13	38304884	*TRPC4*	T	G	0.45	−0.08	0.02	7.73 × 10^−6^	0.99
rs113202356	16	27891845	*GSG1L*	A	G	0.10	−0.14	0.03	8.45 × 10^−6^	-
rs74450576	16	73918519	*-*	A	G	0.04	0.21	0.05	7.40 × 10^−6^	0.80
rs11643897	16	78221370	*WWOX*	C	T	0.32	0.08	0.02	9.70 × 10^−6^	0.87
rs35522803	19	3592734	*GIPC3*	T	C	0.08	−0.15	0.03	3.83 × 10^−6^	0.82
rs60192794	20	52317186	*-*	C	T	0.07	−0.16	0.04	5.66 × 10^−6^	-
**Children**, HELIX (*n* = 1042, nSNP = 6,138,843)										
SNP	Chr	Position	Gene	Effect allele	Other allele	MAF	β	SE	*p*-value	ALSPAC *p*-value
rs7526817	1	28195486	-	A	T	0.09	−0.67	0.15	5.02 × 10^−6^	0.06
rs79810835	1	146999434	-	A	G	0.02	1.37	0.31	6.83 × 10^−6^	-
rs59436870	2	101830050	*TBC1D8*	T	C	0.18	−0.52	0.11	5.16 × 10^−6^	0.74
rs9852537	3	98658085	*CTD-2021J15.1*	T	C	0.03	1.29	0.29	7.62 × 10^−6^	0.66
rs186276942	3	115279279	*-*	G	A	0.01	2.87	0.63	5.29 × 10^−6^	-
rs62287513	3	184104050	*CHRD*	A	G	0.31	0.44	0.09	7.69 × 10^−7^	-
rs28618224	4	21041710	*KCNIP4*	C	A	0.03	1.47	0.32	5.03 × 10^−6^	0.11
rs115812569	4	42726300	*-*	G	A	0.01	1.82	0.40	6.42 × 10^−6^	0.56
rs2904271	4	90284516	*-*	C	A	0.37	−0.41	0.09	1.84 × 10^−6^	0.79
rs113384484	5	151015719	*-*	A	G	0.04	1.03	0.22	1.50 × 10^−6^	0.02
rs79340261	7	35559836	*-*	A	C	0.02	1.93	0.40	1.08 × 10^−6^	-
rs75847252	9	71483481	*PIP5K1B*	C	T	0.02	1.22	0.26	2.96 × 10^−6^	-
rs73510541	11	69313573	*-*	A	G	0.13	0.54	0.12	3.72 × 10^−6^	0.50
rs9510838	13	24317031	*-*	T	G	0.17	0.58	0.12	3.43 × 10^−6^	0.88
rs9563673	13	34239526	*RP11-141M1.3*	C	T	0.07	−0.90	0.19	5.90 × 10^−6^	0.21
rs17106291	14	37559401	*SLC25A21*	G	A	0.02	2.32	0.52	7.53 × 10^−6^	-
rs7154700	14	63201771	*KCNH5*	T	C	0.01	1.61	0.34	2.15 × 10^−6^	-
rs116971963	17	14961821	-	A	G	0.01	1.57	0.34	4.91 × 10^−6^	-
rs145982353	19	54257385	-	G	C	0.03	1.27	0.27	4.07 × 10^−6^	-
rs6075980	20	288233	-	G	A	0.38	0.42	0.09	2.31 × 10^−6^	0.24
rs148653405	20	5594216	-	A	G	0.01	1.77	0.40	8.35 × 10^−6^	0.34

^1^ GRCh37 genomic co-ordinates. ^2^ Variants annotated to genes only if the SNP is in the gene body. ^3^ Minor (effect) allele frequency. ^4^ Standard error. ^5^ “-” indicates the variant was not available in the other study.

**Table 5:** SNP-Hg summary statistics for candidate variants identified in previous studies of Hg or other metal GWAS.

SNP	Gene	Source Study, Direction of Effect, and Biological Matrix	Effect Allele	Other Allele	Study	β	SE	*p*-Value
**Hg—gene association studies**								
rs3811647	*TF*	A allele, lower cord tissue Hg [48]	A	G	ALSPAC	0.00	0.02	0.83
					HELIX	−0.14	0.09	0.11
rs761142	*GCLC*	GG vs. TT, lower hair Hg [107]	C	A	ALSPAC	−0.01	0.02	0.63
					HELIX	0.06	0.10	0.57
rs41307970	*GCLM*	CT + TT vs. TT, lower blood Hg [108]	C	G	ALSPAC	0.00	0.05	0.93
					HELIX	0.20	0.25	0.41
rs10636	*MT2A*	C allele, higher urine Hg [46]	C	G	ALSPAC	0.05	0.02	0.01
					HELIX	−0.06	0.10	0.53
rs2270837 ^1^	*MT1M*	A allele, higher urine Hg [46]	G	A	ALSPAC	0.01	0.03	0.57
rs8052394	*MT1A*	A allele interacts with fish intake for higher hair Hg [46]	G	A	ALSPAC	0.00	0.03	0.96
					HELIX	0.05	0.13	0.69
rs9936741	*MT1M*	T allele interacts with fish intake for higher hair Hg [46]	C	T	ALSPAC	−0.08	0.06	0.17
					HELIX	−0.95	0.37	0.01
**Lead—GWAS**								
rs1805313	*ALAD*	G allele, lower blood lead [54]	G	A	ALSPAC	0.01	0.02	0.57
					HELIX	0.10	0.09	0.25
**Selenium—GWAS**								
rs672413	*ARSB*	G allele, lower blood selenium [56]	G	A	ALSPAC	0.00	0.02	0.92
					HELIX	−0.01	0.09	0.95
rs705415 ^1^	Intergenic	T allele, lower blood selenium [56]	T	C	HELIX	0.15	0.17	0.37
rs6586282	*CBS*	T allele, lower blood selenium [56]	T	C	ALSPAC	−0.03	0.02	0.27
					HELIX	−0.06	0.11	0.61
**Zinc—GWAS**								
rs2120019	*PPCDC*	C allele, lower blood zinc [55]	C	T	ALSPAC	0.00	0.02	0.94
					HELIX	0.07	0.10	0.50
rs1532423	*CA1*	G allele, lower blood zinc [55]	G	A	ALSPAC	−0.03	0.02	0.07
					HELIX	−0.08	0.09	0.36

^1^ Not available in HELIX.

## Data Availability

GWAS summary statistics for LD-clumped SNPs which were below a suggestive *p*-value threshold are reported in Table 2. Full summary statistics for European pregnant women are publicly available on the GWAS Catalog (https://www.ebi.ac.uk/gwas/ (accessed on 1 November 2023)), Study Accession: GCST90271315.

## Supplementary Materials

The following supporting information can be downloaded at: https://www.mdpi.com/article/10.3390/genes14122123/s1, Supplementary Table S1. Summary of study population, measurement methods, and GWAS characteristics. Supplementary Table S2. Previously identified variants. Supplementary Figure S1. Hg distributions. Supplementary Figure S2. GWAS Manhattan plots. Supplementary Figure S3. QQ plots. Supplementary Table S3. Gene mapping of selected results. Supplementary Table S4. Functional analysis of selected results.
