# Supplementary material for: Genome-Wide Association Study of Blood Mercury in European Pregnant Women and Children
Source: Genes (Basel). 2023 Nov 24;14(12):2123. doi: 10.3390/genes14122123 (PMC10742428; doi:10.3390/genes14122123)
Supplement: Supplementary file 1 [file genes-14-02123-s001.zip › genes-2692627-supplementary.pdf]

Supplementary materials: Genome-wide association study of blood mercury in European pregnant women and children

Supplementary Table S1. Summary of study population, measurement methods, and GWAS characteristics.

	**ALSPAC**	**HELIX**
Date of recruitment	April 1991 - December 1992	1999 - 2010
Population	Pregnant women	Children aged 7 to 9
Location	Avon Health Authority area, UK	UK, France, Spain, Lithuania, Norway, and Greece
N recruited	14,833	Approximately 32,000
Mercury sample source	Whole blood	Whole blood
Mercury sample timing	Early pregnancy	6 - 11 years old
Mercury sample analysis method	ICP-DRC-MS	ICP-SFMS
N with mercury sample	4,131	1,301
**Genotyping**		
N with blood DNA sample	10,015	1,397
Genotyping method	Illumina Human660W-Quad Array	Infinium Global Screening Array (GSA) (Illumina)
N SNP directly genotyped	557,124	692,367
QC software	Plink v1.07	Plink v1
Individual missingness	>5%	>3%
SNP missingness	>5%	>5%
Hardy-Weinberg equilibrium	$P < 1.0x10^{-07}$	$P < 1.0x10^{-06}$
Minor allele frequency	<1%	<1%
Cryptic relatedness	IBD > 0.125	IBD > 0.125

Other exclusions	indeterminate X chromosome heterozygocity, extreme autosomal heterozygocity, population outliers	Gender mismatch, heterozygosity, relatedness, non-canonical PAR
N samples post QC	8,196	1,304
N SNP post QC	526,688	509,344
**Imputation**		
Reference panel	Haplotype Reference Consortium (HRC r1.1)	Haplotype Reference Consortium (HRC r1.1)
Phasing	ShapeIt v2	Eagle v2.4
Imputation	Impute V3	minimac4
N SNP after imputation	39,117,141	40,405,505
Minor allele frequency	<1%	<1%
Imputation quality (INFO)	<0.9	<0.9
Hardy-Weinberg equilibrium	$P < 1.0x10^{-07}$	$P < 1.0x10^{-06}$
N SNP post QC	6,649,782	6,143,757
**GWAS**		
N samples mercury + genotype	2,893	1,042
GWAS software	SNPTEST 2.5.2	Plink v1
Covariates	Age + 10 PCA	Age + 10 PCA
Minor allele frequency	<1%	<1%
Other exclusions	Unidentifiable rsid	Unidentifiable rsid

Supplementary Table S2. Previously identified variants.

Variants identified in gene-association studies of mercury or genome-wide association studies of metals that interact with mercury within the body (lead, selenium, zinc).

**SNP**	**Gene**	**Biological sample**	**Population**	**Effect allele**	**Other allele**	**Beta (p-value)**	**PMID**
rs41307970	*GCLM*	Whole blood & plasma	88 adults in Amazon riverside communities.	CT + TT	CC	-0.21 (0.05)	24696865

rs2270837	*MT1M*	Urine	515 dental professionals	A	G	1.85 (0.008)	22233731
rs10636	*MT2A*	Urine	515 dental professionals	C	G	0.06 (-)	22233731
rs8052394	*MT1A*	Hair	515 dental professionals	A * intake	G	-300 (0.02)	22233731
rs9936741	*MT1M*	Hair	515 dental professionals	T * intake	C	19.3 (0.02)	22233731
rs761142	*GCLC*	Hair	1,449 mothers in Seychellois population	GG	TT	-0.46 (0.02)	29573653
rs3811647	*TF*	Cord tissue	1,311 cord samples	A	G	- (0.03)	23903878
**SNP**	**Gene**	**Metal**	**Population**	**Effect allele**	**Other allele**	**Beta (p-value)**	**PMID**
rs1805313	*ALAD*	Lead	5,433 Australian and UK adults.	A	G	+ (3.91 × 10^{-14})	25820613
rs672413	*ARSB*	Selenium	9,639 subjects from meta-analysis of US, Australian, and UK adults	A	G	+ (5.21 x 10^{-14})	25343990
rs705415	-	Selenium	8,054 subjects from meta-analysis of US, Australian, and UK adults	T	C	- (4.64 x 10^{-10})	25343990
rs6586282	*CBS*	Selenium	9,639 subjects from meta-analysis of US, Australian, and UK adults	T	C	- (3.96 x 10^{-09})	25343990
rs1532423	*CA1*	Zinc	2,603 adults Australia	A	G	0.18 (6.40 × 10^{-12})	23720494
rs2120019	*PPCDC*	Zinc	2,603 adults Australia	C	T	-0.29 (1.40 × 10^{-12})	23720494

Supplementary Figure S1. Hg distributions.

Blood Hg concentrations in (a) 2,893 pregnant women (b) 1,042 children aged 6-11 years old.

Includes 7 samples with Hg > 8 µg/L which were removed from Figure 1 for readability.

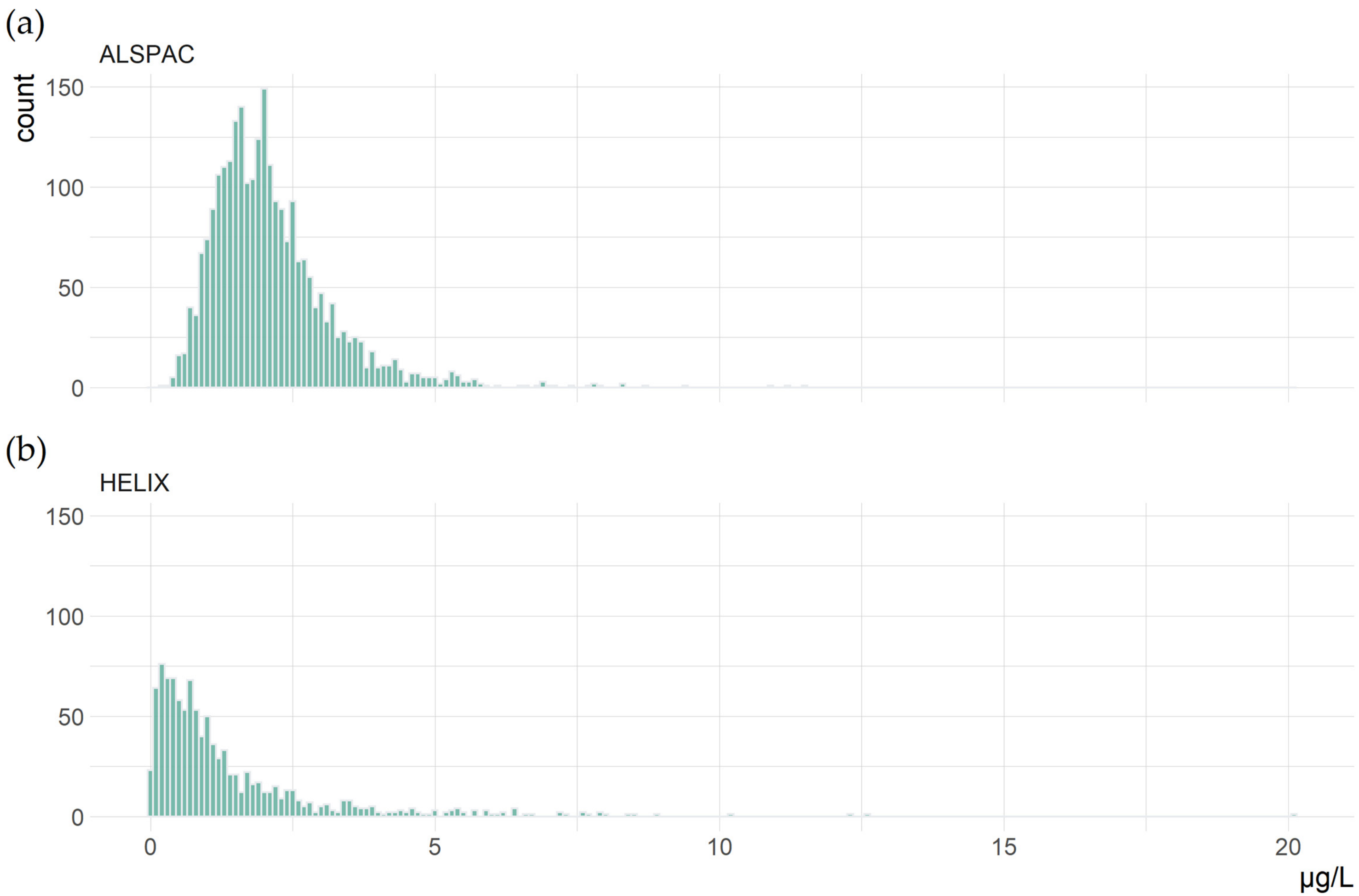

(a)
ALSPAC
count
150
100
50
0
(b)
HELIX
150
100
50
0
0
5
10
15
20
µg/L

Supplementary Figure S2. GWAS Manhattan plots.

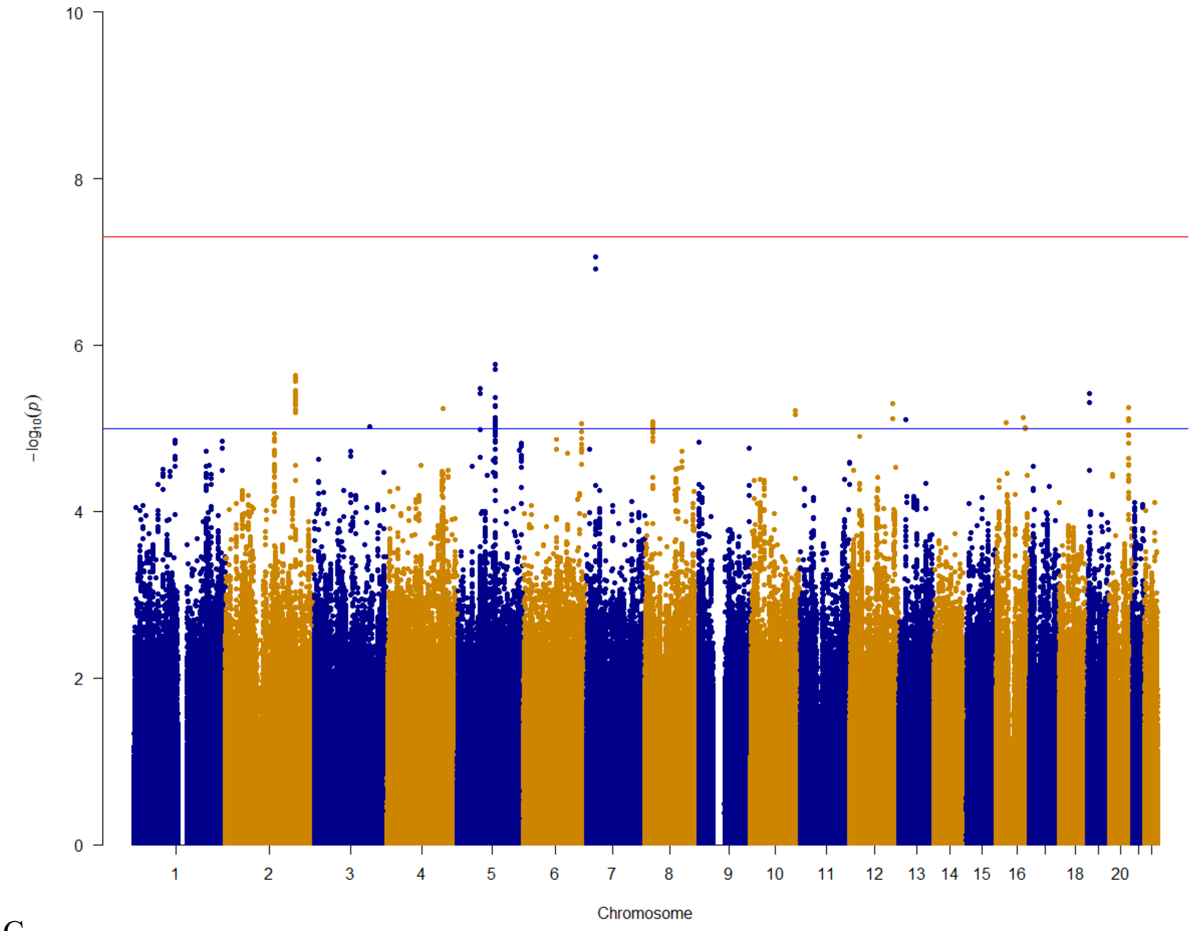

(a) ALSPAC

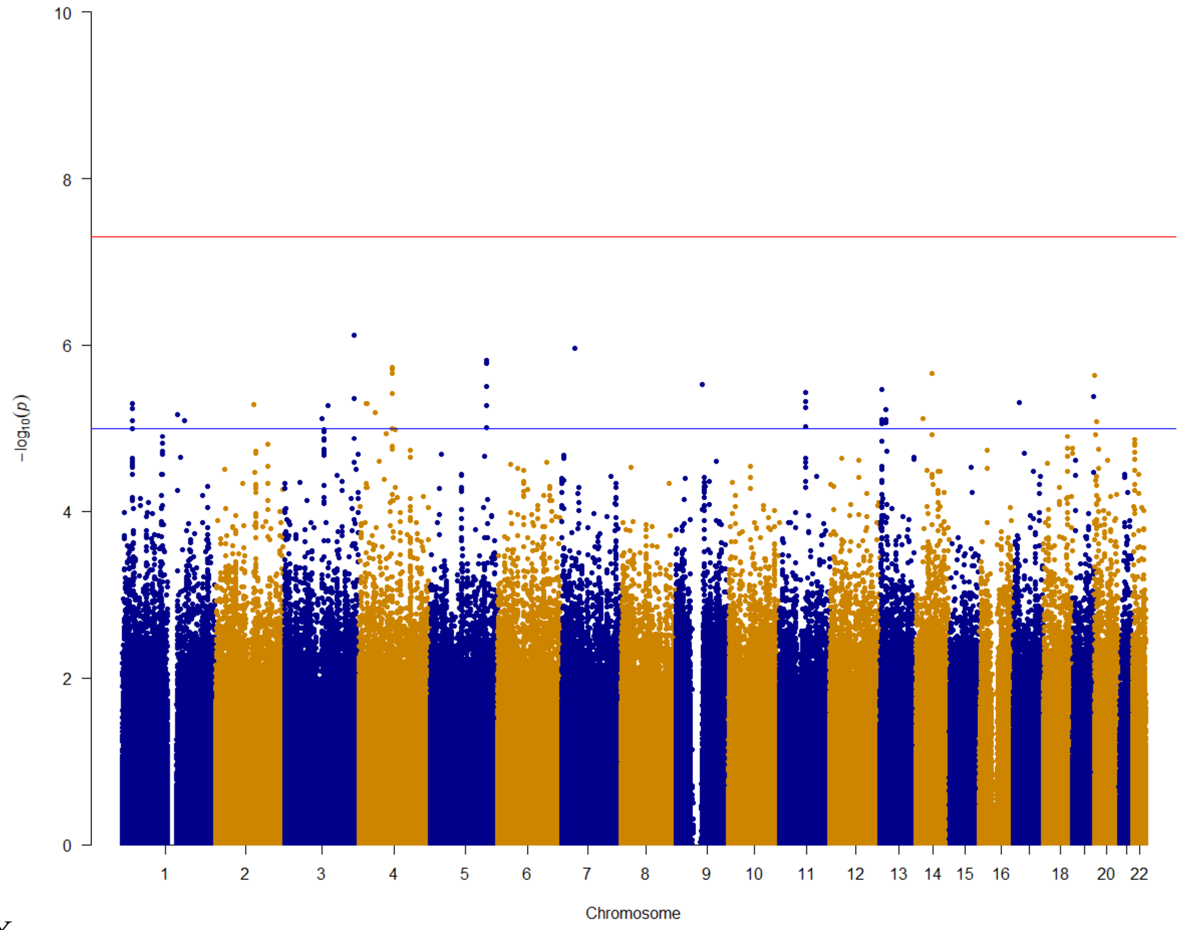
10
8
6
4
2
0
$-\log_{10}(p)$
1
2
3
4
5
6
7
8
9
10
11
12
13
14
15
16
18
20
22
Chromosome

(b) HELIX

Supplementary Figure S3. QQ plots.

Expected and observed p-values

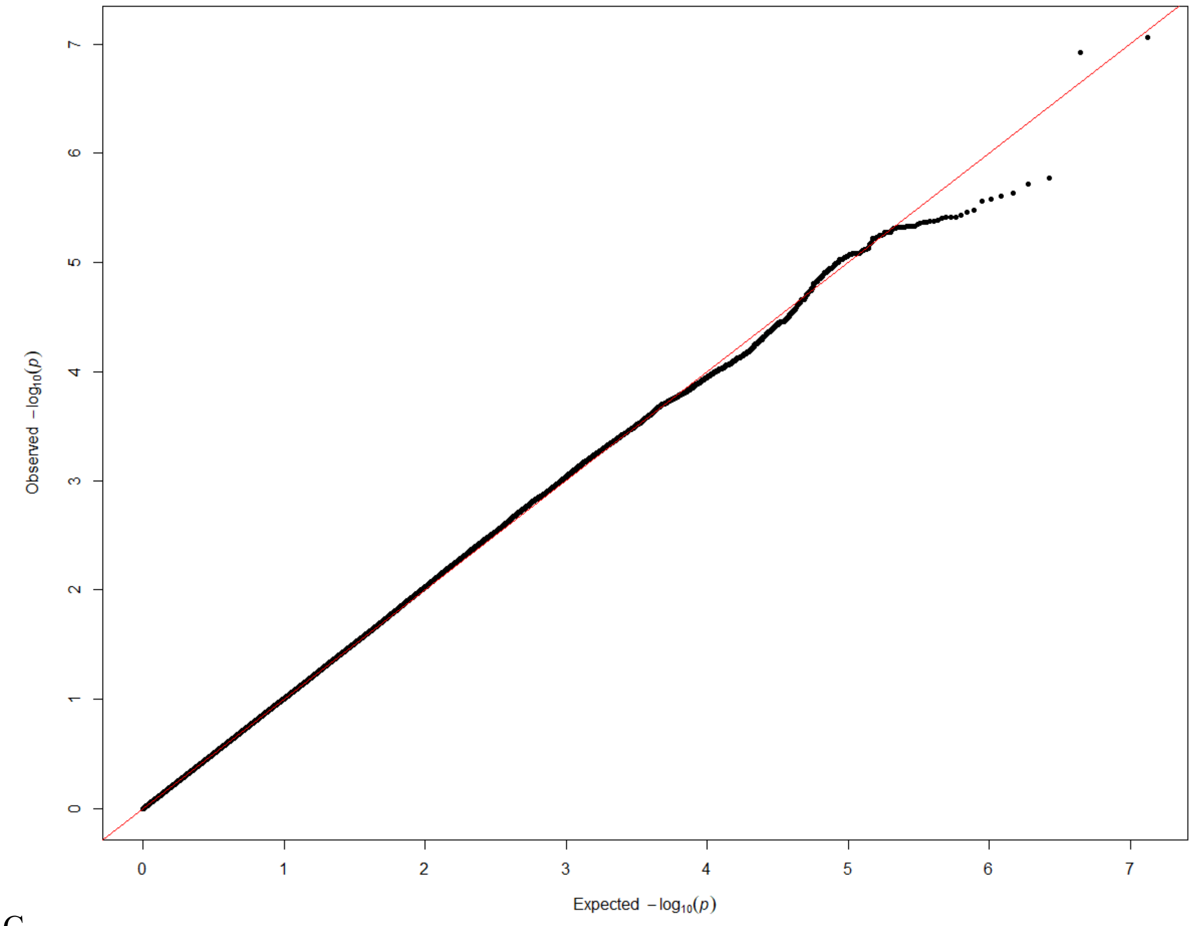

(a) ALSPAC

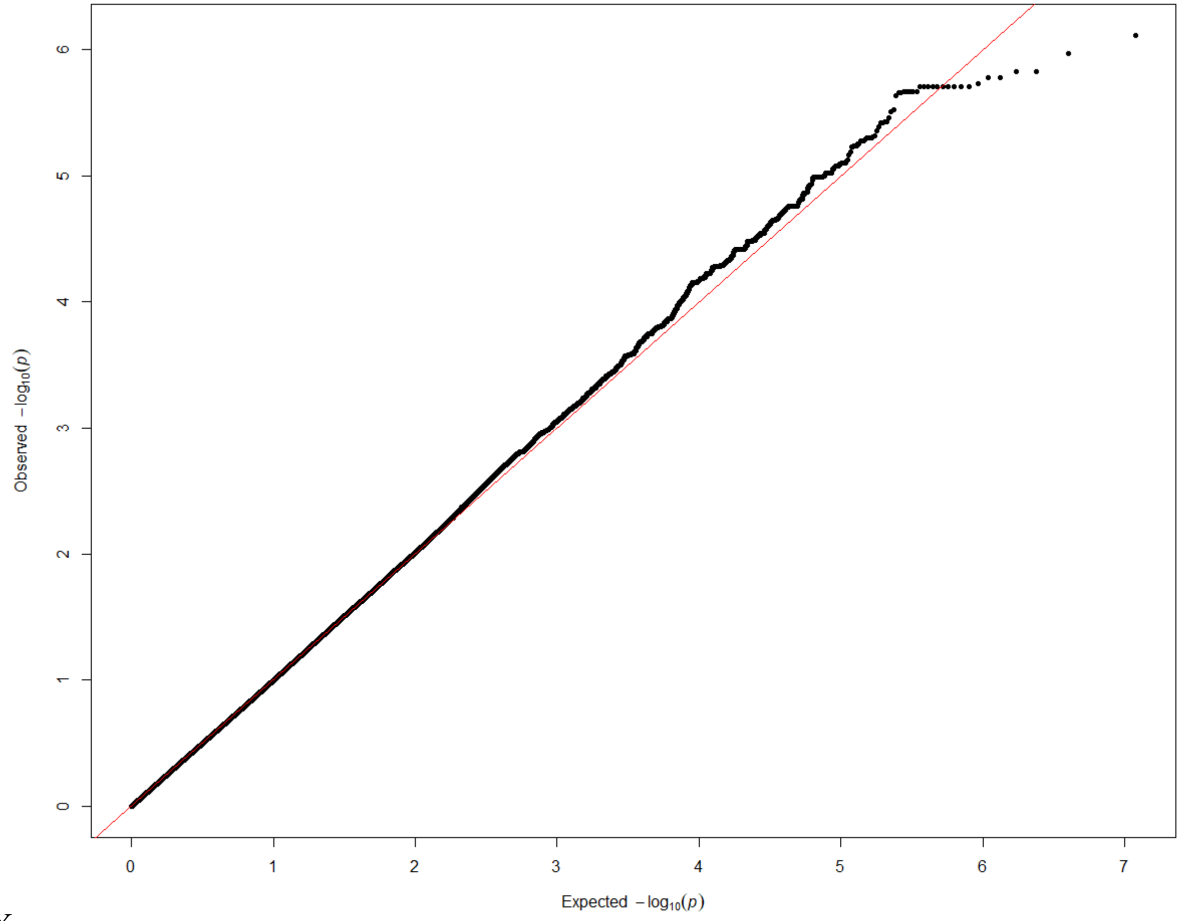

(b) HELIX

Supplementary Table S3. Gene mapping of selected results.

Gene mapping of LD clumped suggestive (p <1x10^{-5}) variants. Automated mapping taken from FUMA SNP2Gene, and manually checked using dbSNP.

(a) ALSPAC

**SNP**	**Chr**	**Position** (GRCH37)	**Gene**	**Distance to lead SNP**	**Type**
rs4853739	2	191698516	*AC005540.3*	47006	intergenic
rs11709754	3	149615531	*RNF13*	0	intronic
rs361166	4	152792791	*GATB*	110632	Intergenic
rs6859392	5	63241481	*RP11-158J3.2*	12238	Intergenic[1]
rs1372504	5	103749428	*RP11-6N13.1*	0	ncRNA_intronic
rs2246509	6	156058999	*RNU7-152P*	82838	intergenic
rs1845418	7	24095415	*RNA5SP228*	76908	intergenic
rs146099921	8	22248665	*SLC39A14*	0	intronic
rs7900717	10	122034207	*RPL21P16*	79950	intergenic
rs7301395	12	116396301	*MED13L*	0	downstream
rs12874443	13	38303606	*TRPC4*	0	intronic
rs113202356	16	27891845	*GSG1L*	0	intronic
rs74450576	16	73918519	*RPSAP56*	56018	intergenic
rs11643897	16	78221370	*WWOX*	0	intronic
rs35522803	19	3592133	*GIPC3*	0	UTR3
rs60192794	20	52317186	*RNU7-14P*	31888	intergenic
1. In linkage disequilibrium with intronic variant within named gene.					

(b) HELIX

**SNP**	**Chr**	**Position** (GRCH37)	**Gene**	**Distance**	**Type**
rs7526817	1	28195486	*THEMIS2*	3568	Intergenic[2]

rs79810835	1	146999434	*LINC00624*	9734	intergenic
rs59436870	2	101830050	*TBC1D8*	0	intronic
rs9852537	3	98658085	*CTD-2021J15.1*	0	ncRNA_intronic
rs186276942	3	115279279	*GAP43*	62891	intergenic
rs62287513	3	184104050	*CHRD*	0	intronic
rs28618224	4	21041710	*KCNIP4*	0	intronic
rs115812569	4	42726300	*ATP8A1*	67178	intergenic
rs2904271	4	90280428	*GPRIN3*	53437	intergenic
rs113384484	5	151015719	*CTB-113P19.4*	16116	intergenic
rs79340261	7	35559836	*AC007652.1*	9962	intergenic
rs75847252	9	71483481	*PIP5K1B*	0	intronic
rs73510541	11	69313573	*AP000439.3*	18864	Intergenic[2]
rs9510838	13	24300617	*MIPEP*	3710	Intergenic[2]
rs9563673	13	34236442	*RP11-141M1.3*	0	ncRNA_intronic
rs17106291	14	37559401	*SLC25A21*	0	intronic
rs7154700	14	63201771	*KCNH5*	0	Intronic[1]
rs116971963	17	14961821	*AC005772.2*	6227	intergenic
rs145982353	19	54257385	*MIR527*	28	downstream
rs6075980	20	288233	*ZCCHC3*	7267	intergenic
rs148653405	20	5594216	*GPCPD1*	2543	Upstream
1. In linkage disequilibrium (r2>0.8) with UTR3 variant within named gene.					
2. In linkage disequilibrium (r2>0.8) with intronic variant within named gene.					

Supplementary Table S4. Functional analysis of selected results.

Summary of functional analysis for LD clumped suggestive ($p< 1x10^{-5}$) variants.

GWAS Catalog: No results for any SNPs.

(a) ALSPAC

SNP	SNP-based search			Gene-based search (results for nearest gene unless otherwise stated)			
	**LDtrait** (r2>0.8)	**Phenoscanner** ($p < 5 \times 10^{-8}$)	**eQTL**	**Gene**	**GeneCards**	**GTEx** (blank: no notable variation)	**OMIM** (human or animal cells)
rs4853739	-	*GLS* & *MFSD6* expression	*GLS* (-0.10, 7.22×10^{-09})	*AC005540.3*[1]	Glutamine hydrolysis catalyst. *MFSD6*: MHC class 1 binding.		-
rs11709754	**-**	*PFN2* expression	*PFN2* (0.16, 4.10×10^{-14})	*RNF13*	Protein-protein interactions, specifics unknown	Brain, whole blood	-
rs361166	Trans fatty acid levels	Histone modification, educational attainment	-	*GATB*[1]	Glutaminyl-tRNA synthetase, involved in glutamine transfer	Brain, heart, skin	-
rs6859392	-	-	-	*RP11-158J3.2*[2]	-	Brain - hippocampus and front cortex	-
rs1372504	Major depressive disorder, insomnia	Daytime napping, doctor visit for anxiety/depression.	-	*RP11-6N13.1*	-	Testis	-
rs2246509	-	-	-	*RNU7-152P*[1]	-	Brain - amygdala	-
rs1845418	-	-	-	*RNA5SP228*[1]	-	Kidneys	-

rs146099921	-	-	*SLC39A14* (-0.16, 6.84×10^{-09})	*SLC39A14*	Metal transporter, cellular uptake of cadmium, iron, manganese, zinc	Arteries, liver, pancreas	Transport and homeostasis of zinc, manganese, and iron.
rs7900717	-	-	-	*RPL21P16*[1]	-	-	-
rs7301395	-	-	-	*MED13L*	Mediator component between regulatory proteins and RNA transcription.	-	-
rs12874443	-	-	-	*TRPC4*	Calcium ion channels	Arteries, uterus	-
rs113202356	-	-	-	*GSG1L*	Synapse receptor activity	Arteries, brain	-
rs74450576	-	-	-	*RPSAP56*[1]	-	-	-
rs11643897	-	-	-	*WWOX*	Tumour suppression	-	-
rs35522803	-	*GIPC3* expression	-	*GIPC3*	Hair bundle/cell maturation.	-	-
rs60192794	-	-	-	*RNU7-14P*[1]	-	Testis	-
1. Intergenic variant, gene listed is nearest.							
2. Located outside of gene, and in linkage disequilibrium (r2>0.8) with variant within gene.							

(b) HELIX

**SNP**	**SNP-based search**			**Gene-based search (results for nearest gene unless otherwise stated)**			
	**LDtrait** (r2>0.8)	**Phenoscanner** ($p < 5 \times 10^{-8}$)	**eQTL**	**Gene**	**GeneCards**	**GTEx** (blank: no notable variation)	**OMIM** (human or animal cells)
rs7526817	**-**	*RPA2* expression	*THEMIS2* (0.9, 6.10×10^{-10})	*THEMIS2*[2]	T cell receptor signalling	Whole blood	-
rs79810835	**-**	*ACP6* expression		*LINC00624*[1]	-	Testis	-

rs59436870	-	*SNORD89* expression	*RNF19* (0.57, 3.15 x 10-28)	*TBC1D8*	GTPase activator activity, predicted to be involved in intracellular protein transport *SNORD89*: ovarian cancer progression. *RNF19:* Ubiquitin ligase protein activity.	-	-
rs9852537	-	Type 2 lactosamine alpha-2,3-sialyltransferase	-	*CTD-2021J15.1*	-	-	-
rs186276942	-	-	-	*GAP43*[1]	Neuronal growth	Brain, nerve	-
rs62287513	-	-	-	*CHRD*	Early vertebrate embryonic development	Ubiquitous - high in brain	-
rs28618224	-	-	-	*KCNIP4*	Potassium voltage-gated channels	Brain	-
rs115812569	-	-	*ATP8A1* (0.34, 8.29×10^{-08})	*ATP8A1*[1]	Lipid (phospholipids) transport across membranes	Brain, thyroid	-
rs2904271	-	-	-	*GPRIN3*[1]	Neuron development.	Brain and lungs	-
rs113384484	-	*SPARC* expression	-	*CTB-113P19.4*[1]	Bone collagen calcification.	Oesophagus, skin, brain.	-
rs79340261	-	-	-	*AC007652.1*[1]	-	Testis	-
rs75847252	-	*TJP2* expression.	-	*PIP5K1B*	Regulation of lipid messenger.		-
rs73510541	-	-	-	*AP000439.3*[1]	-	Small intestines, colon, skin	-

rs9510838	-	-	-	*MIPEP*[1]	Maturation of oxidative phosphorylation-related proteins	-	-
rs9563673	-	-		*RP11-141M1.3*	-	-	-
rs17106291	-	Treatment with antihypertensive	-	*SLC25A21*	Transports dicarboxylates across the inner membranes of mitochondria and participates in lysine, tryptophan, and hydroxylysine catabolism	Testis	-
rs7154700	-	Cause of death: alcohol hepatitis.	-	*KCNH5*	Potassium voltage-gated channels	Brain	-
rs116971963	-	-	-	*AC005772.2*[1]	-	Testis	-
rs145982353	-	-	-	*MIR527*	Micro RNA	-	-
rs6075980	-	-	-	*ZCCHC3*[1]	DNA and RNA binding	-	-
rs148653405	-	-	-	*GPCPD1*[2]	Glycerophospholipid catabolic process	-	-
1. Intergenic variant, gene listed is nearest.							
2. Intergenic variant, and in linkage disequilibrium (r2>0.8) with variant within gene.							
